# Characterization of the pattern of expression of Gas1 in the kidney during postnatal development in the rat

**DOI:** 10.1371/journal.pone.0284816

**Published:** 2023-04-24

**Authors:** Andrea Cetina-Palma, Carmen Namorado-Tónix, Rafael Rodríguez-Muñoz, Paula Vergara, José Luis Reyes-Sánchez, José Segovia

**Affiliations:** 1 Departamento de Farmacología, Centro de Investigación y de Estudios Avanzados del IPN, Mexico City, Mexico; 2 Departamento de Fisiología, Biofísica y Neurociencias, Centro de Investigación y de Estudios Avanzados del IPN, Mexico City, Mexico; Khalifa University of Science and Technology, UNITED ARAB EMIRATES

## Abstract

Growth Arrest-Specific 1 (Gas1) is a pleiotropic protein with different functions, in the adult kidney Gas1 acts as an endogenous inhibitor of cell proliferation but it is also necessary for the maintenance and proliferation of Renal Progenitor Cells (RPC) during early development, thus it fulfills important functions in the adult kidney. However, it is not known whether or not Gas1 is expressed during postnatal development, a critical stage for renal maturation. For this reason, the main objective of this work was to characterize the expression pattern of Gas1 in the different regions of the kidney by immunofluorescence and Western blot analysis during the postnatal development of the rat. We found that Gas1 is present and has a differential expression pattern in the various regions of the nephron during postnatal development. We observed that the highest levels of expression of Gas1 occur in the adult, however, Gas1 is also expressed in RPC and interestingly, the expression of RPC markers such as the Neural cell adhesion molecule (NCAM) and Cluster of differentiation 24 (CD24) were found to have an inverse pattern of expression to Gas1 (decreases as the kidney matures) during postnatal renal maturation, this indicates a role for Gas1 in the regulation of renal cell proliferation at this stage of development.

## Introduction

At the microscopic level, the nephron is the structural and functional unit of the kidney [1]. The different stages of development (embryonal, postnatal, maturity and old age) involve a strict integration and control of various biological processes, such as: proliferation, differentiation, and cell death. It has been demonstrated that Gas1 inhibits cell growth [2–4]. Gas1 has also been reported to interact with the Glial cell-derived neurotrophic factor (GDNF) [5, 6], and the Sonic Hedgehog (Shh) [7], two critical molecules during central nervous system (GDNF/Shh) and renal (GDNF) development, suggesting a role for Gas1 as a developmental regulator. Additionally, it is known that Gas1 is expressed in different types of progenitor cells [8–10]. At the renal level, previous studies have demonstrated that Gas1 is expressed in the kidneys of healthy mice [11], in podocytes of healthy adult rats and that glomerular mesangial cells were capable of secreting Gas1 *in vitro*, evidencing that Gas1 could be acting as an endogenous inhibitor of cell proliferation [12]. It has also been reported that Gas1 is expressed in Bowman’s capsule in the healthy kidney of adult rats [13].

New nephrons (nephrogenesis) are generated from RPCs during embryonic development and interestingly, in some mammals, such as rats, it ends during postnatal development [14]. There are reports showing a key role of Gas1 in the maintenance of RPC during embryonic renal development [15], however, there are no reports describing whether Gas1 is expressed in the kidney during the next stage, postnatal development. It is also important to highlight that during this stage renal maturation takes place [16–19]. During this period, various morphological and functional changes occur at the renal level [20] and the cells multiply rapidly [21], so the participation of agents that regulate these events is essential.

Gas1 is a pleiotropic protein [22] and its presence is necessary for the maintenance and proliferation of RPCs during early development of the kidney [15], and since RPCs are essential during postnatal development, we hypothesized that Gas1 is expressed during the postnatal development of the kidney. Therefore, the main objective of this work was to explore the expression of Gas1 during postnatal development of the rat, to identify the regions of the nephron where it is expressed and to determine whether there is any relationship between Gas1 and RPCs.

## Materials and methods

### Antibodies and reagents

The following primary and secondary antibodies were used for Western blot and/or immunostaining analysis: anti-Gas1 produced by Pro-Sci (Poway, CA, USA) [23–25], anti-claudins-2, -4, -8 (Cat. #32–5600, 32–9400, 40-0700Z); anti-Alexa Fluor 594 (Cat. #A-21442, A-11062), anti-Alexa Fluor 488 (Cat. #A-11055, A-11001) and anti-alpha tubulin (Cat. #32–2700) were obtained from Invitrogen (Carlsbad, CA, USA). Anti-CD24 antibody (Cat. #551133) was obtained from BD Pharmingen (San Diego, CA, USA). Anti-NCAM, anti-AQP2, anti-Nephrin and anti-Dendrin antibodies (Cat. #sc-1507, sc-9882, sc-19000, sc-167616) were obtained from Santa Cruz Biotech, Inc. (Dallas, TX, USA). Anti-dipeptidyl peptidase IV (DppD) antibody (Cat. #MCA924R) was obtained from AbD Serotec (Raleigh, NC, USA) and anti-actin was a gift from Dr. Jose Manuel Hernandez (Department of Cell Biology, Cinvestav-IPN) [26].

### Experimental model and tissue collection

This study was carried out in strict accordance with the recommendations of the Guide for the Care and Use of Laboratory Animals of the Mexican Official Norm NOM-062-ZOO-1999. The protocol was approved by the Committee on the Ethics of Animal Experiments of the Institutional Animal Care Committee (UPEAL). Experimental subjects were female Wistar rats at different postnatal days (PND), at PND 1, 3, 7, 10, 14, 21 and 2-month-old adult (ADL). Rats were maintained in a normal 12/12 h light/dark cycle at an average temperature of 22 ± 1°C and 50 ± 5% humidity. Animals had *ad libitum* access to food and water. All efforts were made to minimize suffering. As a method of euthanasia, we use decapitation for newborn and neonate rats up to 14 PND. For 21 PND and adult rats, we injected pentobarbital intraperitoneally and later cervical dislocation was performed. Rats were sacrificed at the indicated PND, and blood, urine, and kidney tissue samples were collected. The kidneys of each PND were perfused with saline (0.9% NaCl) and then placed in cold saline. Subsequently, they were cryopreserved by immersing them in 2-methyl-butane (Aldrich; Milwaukee, WI) for 30 sec, followed by 2.5 min in liquid nitrogen and then at room temperature for another 2.5 min (only PND 1, 3, 7, 10, 14 and 21). For kidneys of the ADL group, sections with an approximate thickness of 0.3 mm were cut and immersed in 2-methyl-butane for 1 min, followed by 5 min in liquid nitrogen and then at room temperature for an additional 5 min. Finally, the tissue was stored at -80°C.

### Physiological and biochemical parameters

Urine and blood samples were obtained from each experimental group. Blood samples were obtained by cardiac puncture and centrifuged to obtain serum. The proteinuria analysis was performed using the Lowry method (Bio-Rad Protein Assay Kit, Bio-Rad Laboratories, CA, USA) and creatinine clearance using the Jaffe reaction as previously described [27, 28]. Urine and serum samples were stored at -80°C prior to use. All experiments were performed in triplicate and at different times.

### Immunofluorescence

Kidney tissue cryopreserved in 2-methyl-butane was sectioned (6 μm thick) and placed on previously gelatinized slides. The protocol to determine Gas1 or any other protein in the glomerulus is different compared to tubular structures, thus we used different protocols for each section of the nephron. Immunofluorescence was performed as previously described [29, 30] with the appropriate modifications depending on the region, as previously indicated. Glomerular region of the nephron: sections were fixed with 4% PFA with 30% sucrose (Aldrich M3, 263–1; Milwaukee, WI, USA) at 4°C, and incubated with sodium citrate buffer (JT Baker, Xalostoc, México) 10 mM pH 6.0 at 80°C for 20 min and subsequently incubated with sodium citrate buffer at room temperature for 20 min. Sections were permeabilized with PBS Tween 20% 1X for 10 minutes. Then they were blocked with 0.5% free IgG albumin (1331-A, Research Organics, Cleveland, OH, USA) for 1 h at room temperature and then incubated at 4°C overnight in the presence of the primary antibodies against NCAM (1:250), Nephrin (1:250) and Gas1 (1:500), respectively. Distal tubular region: sections were fixed with methanol for 10 min at 4°C and permeabilized with Triton X-100 at 0.2% for 5 min at room temperature, then blocked with 0.5% IgG-free albumin for 1 h at room temperature and incubated overnight at 4°C with primary antibodies against NCAM (1:250), Cln4 (1:250), CD24 (1:250), and Gas1 (1:500), respectively. Proximal tubular region: sections were fixed with methanol for 10 min at 4°C, slides were incubated with sodium citrate buffer (J. T. Baker, Xalostoc, México) 10 mM pH 6.0 at 4°C for 20 min and permeabilized with PBS Tween 20 1% for 14 min at 4°C. Subsequently, sections were blocked with 0.5% IgG-free albumin for 1.5 hrs. at room temperature and incubated overnight at 4°C with the primary antibodies against NCAM (1:250), DppD (1:250), CD24 (1:250) and Gas1 (1:500) respectively. Isolated proximal tubules: Proximal convoluted tubules were obtained manually as previously described [31]. Tubules were fixed for 1 min in ethanol and then in acetone at room temperature. They were incubated in 10 mM sodium citrate buffer pH 6.0 at 4°C for 20 min, then permeabilized with PBS Tween 20 1% for 10 min and blocked with 0.5% free of IgG albumin for 1 h at room temperature and incubated at 4°C overnight in the presence of primary antibodies against Cln2 (1:250) and Gas1 (1:500). The Alexa Fluor 594 anti-rabbit (red, 1:600) and Alexa Fluor 488 anti-goat/anti-mouse (green, 1:600) secondary antibodies were used. Images were acquired with an inverted confocal microscope (TCS-SP8, Leica, Heidelberg, Germany) and analyzed with the LAS AF LITE program (Leica Microsystem). Immunofluorescence experiments were performed with samples from more than 3 animals from each group studied.

### Isolation of proximal tubules for immunofluorescence

Rat tubules were isolated as previously described [32]. Briefly, after sacrifice, kidneys were quickly obtained and placed at 4°C in solution containing (mmol/L): 115 NaCl, 25 NaHCO_3_, 3.5 KCl, 1.25 KH_2_PO_4_, 1.25 CaCl_2_, 1.25 MgSO_4_, 5.5 dextrose, 1.0 sodium citrate, 1.0 sodium lactate, 1.12 l-alanine, 0.9 glycine, 1 g albumin (Sigma Co., St. Louis, MO, USA). Sections of 1–2 mm from the corticomedullary region of the kidney were obtained and placed in glass Petri dishes immersed in the previously described solution at 4°C and pH 7.4. The tubules were identified and dissected manually using a stereomicroscope (X20).

### Isolation of glomerulus

Glomeruli were isolated using the mechanical sieving technique previously described [30]. Briefly, the cortex of the kidneys previously cryopreserved were obtained, and homogenized. The homogenate was pushed through a stainless-steel sieve (117 μm pore, cat. N° 8321A44; Thomas Scientific, Swedesboro, NJ, USA). The material that remained in the upper part of the sieve (enriched in glomeruli) was collected with a 1X solution of Krebs bicarbonate (KB, mM) (NaCl 110 mM, NaHCO_3_ 25 mM, KCl 3 mM, CaCl_2_ 1.2 mM, MgSO_4_ 0.7 mM, 2 mM KH_2_PO_4_, 10 mM sodium acetate, 5.5 mM glucose, 5 mM alanine and 0.5 g/L bovine serum albumin, pH 7.4), at 4°C and centrifuged at 20,000 × g for 10 min. The glomerulus-enriched suspension was transferred to a second sieve (74 μm pore, cat. No. 8321A58; Thomas Scientific). After several washes with PBS at 4°C, the material that remained in the upper part of the sieve contained the glomeruli. It was collected in PBS at 4°C and centrifuged at 20,000 × g for 10 min. The total protein content in glomerulus lysates was determined using Lowry’s method. To confirm the isolation of the glomeruli, the Dendrin protein was used as a positive control in the Western blot analysis.

### Percoll gradient method

Enriched fractions of distal and proximal tubules were obtained from the renal cortex by Percoll density gradient centrifugation as previously described [28, 31]. Briefly, once the kidneys were obtained, they were placed in KB solution, at 4°C. Cortical fragments were obtained and digested with collagenase. Subsequently, samples were transferred to an Erlenmeyer flask previously covered with silicone (prevents adhesion of the tissue to the walls). The tissue in suspension was gassed with carbogen for 30 min at 37°C and with constant agitation. The enzymatic digestion was stopped with KB solution at 4°C. The suspension was filtered (through a metal mesh) to remove collagen fibers and resuspended in 10 ml of 1X KB solution at 4°C; centrifuged at 20,000 × g for 20 min and the pellet resuspended in KB solution at 4°C, this step is repeated two more times. Subsequently, the tissue was resuspended in 5% albumin for 5 min at 4°C and centrifuged again. The pellet was resuspended in the Percoll solution (1:1) and centrifuged at 4°C for 30 min at 20,000 × g, establishing a gradient that separates the tissue into three bands: band 1 (b1), distal tubules; band 2 (b2), mixed tubules and band 3 (b3), proximal convoluted tubules. Total protein content in distal (b1) and proximal (b3) lysates was determined by Lowry’s method. To confirm the enrichment of distal and proximal tubules, claudin (Cln) 4 and 8 proteins; and SGLT1 and claudin (Cln) 2 respectively, were used as positive controls for Western blot analysis.

### Protein extraction

We performed the extraction of total proteins from the glomerular isolate and from the Percoll b1/b3 and incubated each sample for 30 min at 4°C in lysis buffer (RIPA; 10 mM) containing: 40 Tris-HCl, pH: 7.6, 150 NaCl, EDTA, 10% glycerol, 1% Triton X-100, deoxychloride 0.5% sodium and SDS. Subsequently, samples were sonicated 3 times for 30 sec with a high intensity ultrasonic processor (Vibra cell; Sonics & Materials Inc., Danbury, CT, USA) and centrifuged at 4°C for 20 min, and the supernatants were recovered. Quantification of the total protein was carried out with the Lowry method using the Micro BCA Protein Assay Reagent Kit (Pierce, Rockford, IL, USA).

### Western blot analysis

Protein samples were denatured and loaded (45 μg) on 12% SDS-PAGE gels. Gels were run at 90 V for 150 min and the proteins were transferred to polyvinylidene fluoride membranes (PVDF, Millipore Corp. Bedford, MA, USA). Subsequently, nonspecific protein binding was blocked with 1% casein and incubated at 4°C overnight with the primary antibodies, Gas1 (1:500), Dendrin (1:500), NCAM (1:300), actin (1:1000), Cln2 (1:250), SGLT1 (1:500), AQP2 (1:300), α-tubulin (1:1000), CD24 (1:250), Cln4 (1:250), Cln8 (1:250) and DppD (1:500). As a positive control for the Gas1 protein, we used a sample of the cerebellum (CBM) [3]. Proteins were detected with a secondary antibody conjugated to peroxidase (1:10,000) at room temperature with chemiluminescence (UVP Biomaging Systems, Cambridge, UK). The quantification was carried out by measuring the signal intensity of the protein band with the Image J program (National Institute of Health, Bethesda, MD, USA). Each PND analyzed is expressed as the relative density of a group of 20 (younger ages) and 8 (21 PND and adult) rats normalized with actin/α-tubulin as loading control.

### Pixel quantification

For the analysis of semi-quantification of pixels, a threshold segmentation was carried out and a binarization system was applied to all analyzed images [13, 33]. Quantification was performed on high-quality images captured with the inverted confocal microscope (TCS-SP8, Leica, Heidelberg, Germany) and analyzed with the Leica LAS AF Lite software. The pixel quantification values were obtained by taking the relationship between the value of the total pixels in a specific area. Several fields per sample and several samples (n>3) were analyzed for each experimental condition.

### Pearson correlation coefficient and the Mander’s overlap coefficient

We used the Pearson’s correlation coefficient as a statistical test to evaluate the magnitude of the association or correlation between two proteins. The coefficient values range from +1 to -1, where +1 indicates a total positive colocalization, -1 a total negative colocalization and 0 that there is no colocalization between the proteins studied. The Manders’ overlap coefficient ranges between 0 and 1, with 0 corresponding to pixels that do not overlap and 1 to pixels with 100% overlap [34].

### Data analysis

All results are shown as means ± standard deviation. For the comparison of two groups, the Student’s t test was used. Statistical differences between groups were analyzed by one-way analysis of variance (ANOVA) and the Tukey test was used as a post-hoc test. Data was analyzed using GraphPad Prism 6.0 software (GraphPad Software, Inc., La Jolla, CA, USA). A p<0.05 was considered statistically significant.

## Results

### Renal function

**Fig 1:**
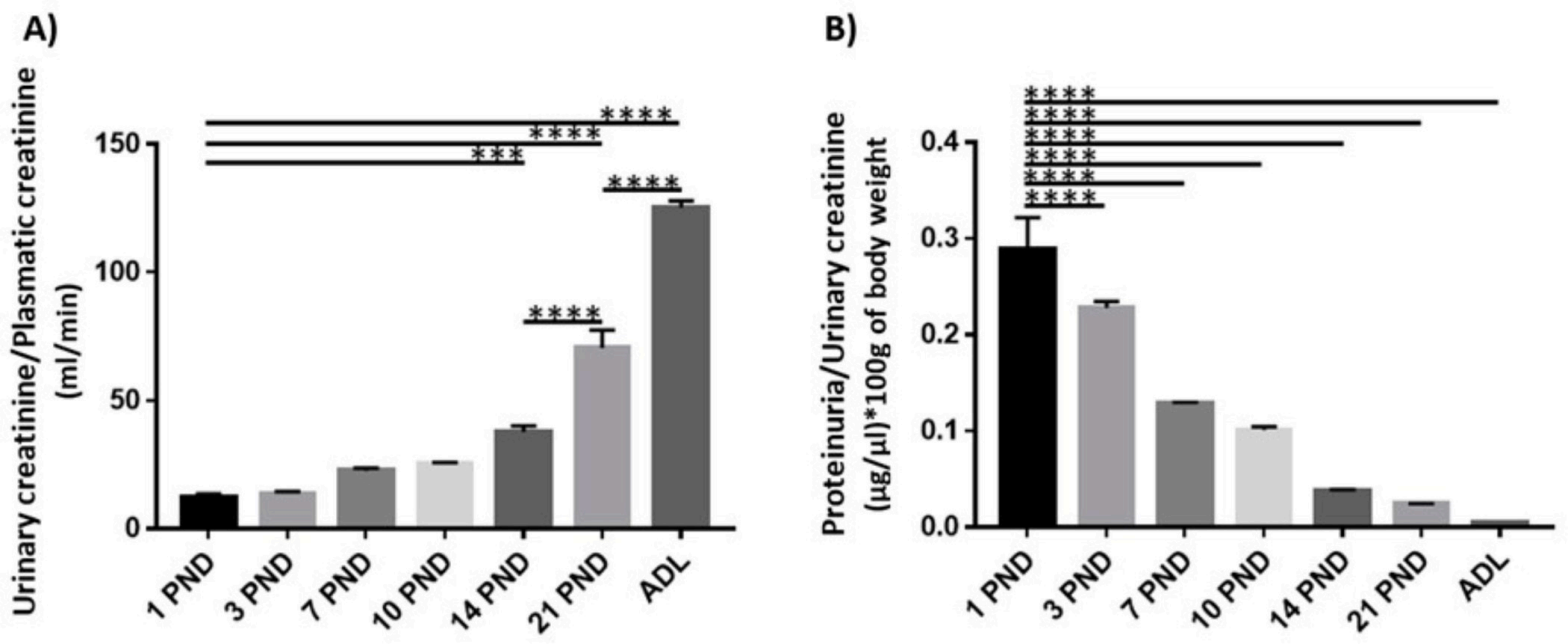
Physiological parameters of rats during postnatal development and in adulthood. A) analysis of the relation between urinary creatinine concentration and serum creatinine, finding a significant and progressive increase as the kidney matures. B) proteinuria analysis, we found the highest values in the first postnatal days (PND), decreasing significantly during renal maturation. Values are means ± standard deviation (SD). One-way ANOVA. *p<0.05, **** p<0.0001. A representative experiment is shown (n = 3).

To demonstrate the postnatal renal maturation process, we analyzed two parameters of renal function: glomerular filtration, and proteinuria [18]. We evaluated the relationship between urine creatinine concentration and serum creatinine concentration, that is, the U/P ratio (Fig 1A) and found a progressive and significant increase in glomerular filtration from the time rats were born to adulthood. This is best observed if we compare PND 1 with PND 14, 21 and ADL, showing that renal function increases as the postnatal days advance and therefore, as the kidney matures. The second parameter that we analyzed was proteinuria, in Fig 1B we can see that in the newborn rat there is a high concentration of protein in the urine that gradually and significantly decreases as the kidney matures, presenting minimal proteinuria in the adulthood.

### Gas1 is expressed in the glomerulus during postnatal renal development and in the adulthood

**Fig 2:**
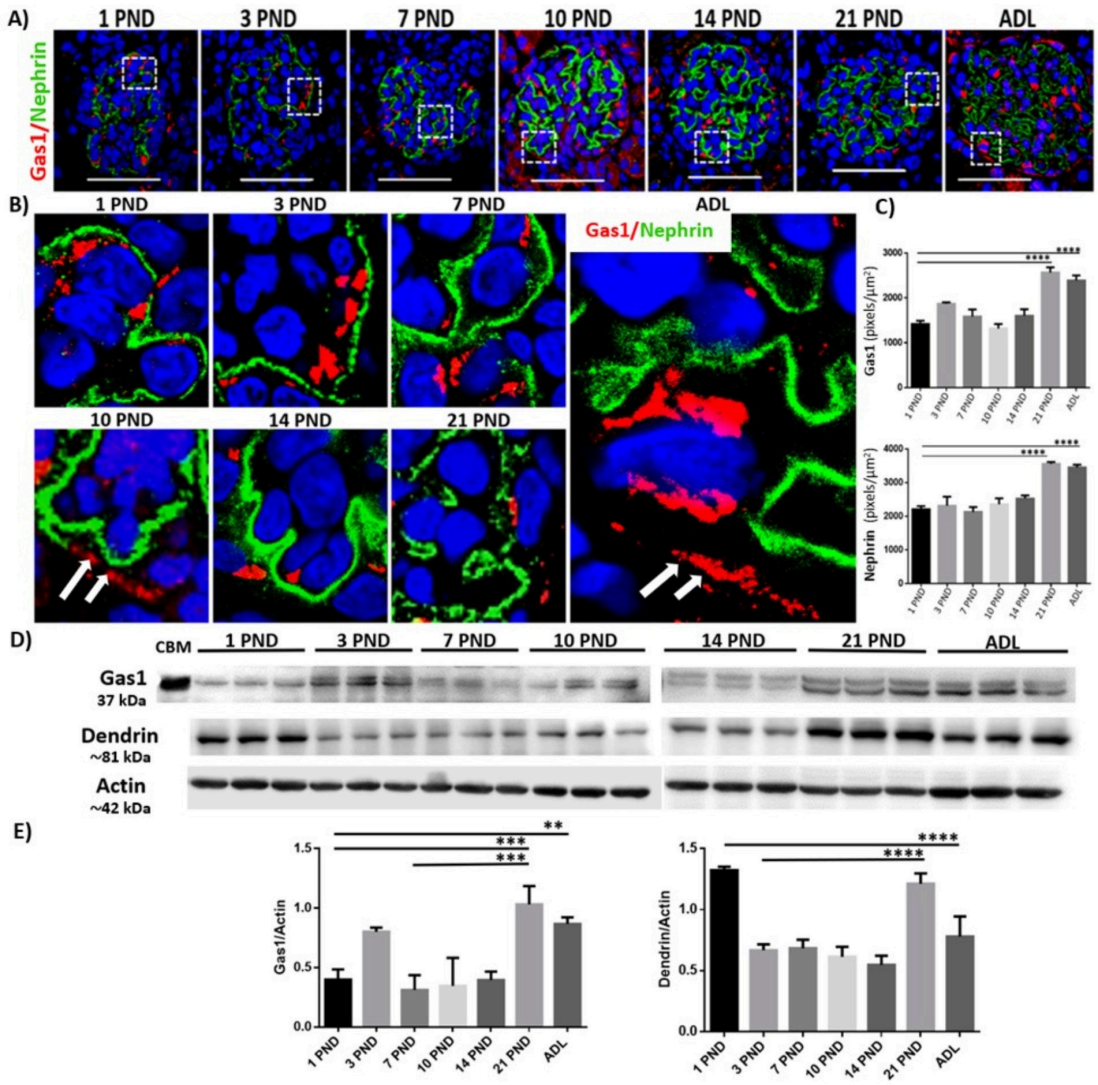
Gas1 is expressed in the glomerulus during postnatal renal development and in adulthood in the rat. A) Immunofluorescence of the general expression pattern of Gas1 and Nephrin on the different postnatal days (PND) evaluated. Gas1 is expressed in the glomerulus and Bowman’s capsule, and its expression increases as the kidney matures. B) magnification of the boxes in A, Gas1 is expressed intraglomerularly and in Bowman’s capsule (BC) (white arrows). C) Semi-quantitative analysis of Gas1 and Nephrin in the glomerulus, a significant increase in the expression of both proteins is observed during renal maturation (PND 21 and ADL). D and E) analysis of the expression of Gas1 and Dendrin by Western blot in an enriched sample of glomeruli. We used a cerebellum sample (CBM) as a positive control for Gas1 expression. Both bands of Gas1 were considered for the densitometric analysis. Each PND analyzed is expressed as the relative density of a group of 20 (younger ages) and 8 (21 PND and adult) rats normalized with actin as loading control. Gas1 (red), Nephrin (green) and nuclei with DAPI (blue). The white squares represent the magnification of specific areas in the glomerulus. Scale bars = 50 μm. Values are means ± standard deviation (SD). One-way ANOVA. *p<0.05, **** p<0.0001. Immunofluorescence experiments were performed in kidney sections. A representative experiment is shown (n = 3).

Due to the limited information regarding the expression of Gas1 during postnatal period in the rat, we evaluated whether it was expressed in the glomerulus and subsequently, its expression pattern during postnatal development and therefore, in renal maturation using immunofluorescence and Western blot analysis. To delimit the glomerular region, we used Nephrin and Dendrin as specific markers of the glomerulus [13, 35]. Gas1 is present from PND 1, its expression is intraglomerular and it is maintained as the kidney matures. The pattern of Gas1 expression during postnatal development is shown in Fig 2A. Interestingly, we found that Gas1 is expressed in Bowman’s capsule (BC) from PND 10 and maintains a permanent and homogeneous expression in BC until the adult stage (Fig 2B, white arrows), suggesting a possible role of Gas1 as a marker of cell differentiation at the glomerular level. Since Gas1 did not co-localize with Nephrin at any stage of development, we suggest that Gas1 is not expressed in the diaphragm slit. Using Western blot analysis of an enriched glomerular fraction, we confirmed that Gas1 has a dynamic expression pattern throughout postnatal renal development, significantly increasing its expression in the mature kidney (PND 21 and ADL, Fig 2D and 2E). This was also observed with a semi-quantitative analysis, the highest level of expression of Gas1 and Nephrin is in the mature kidney (Fig 2C). We also found a double band of Gas1 visible from PND 3 (Fig 2D). In contrast, Dendrin expression has a differential expression pattern during postnatal development since its highest level of expression is at PND 1 and subsequently decreases, and significantly increasing again at PND 21 and ADL (Fig 2D and 2E).

### Gas1 is expressed in renal progenitor cells in the glomerulus and Bowman’s capsule and has an inverse expression pattern during postnatal renal development and in the adulthood

**Fig 3:**
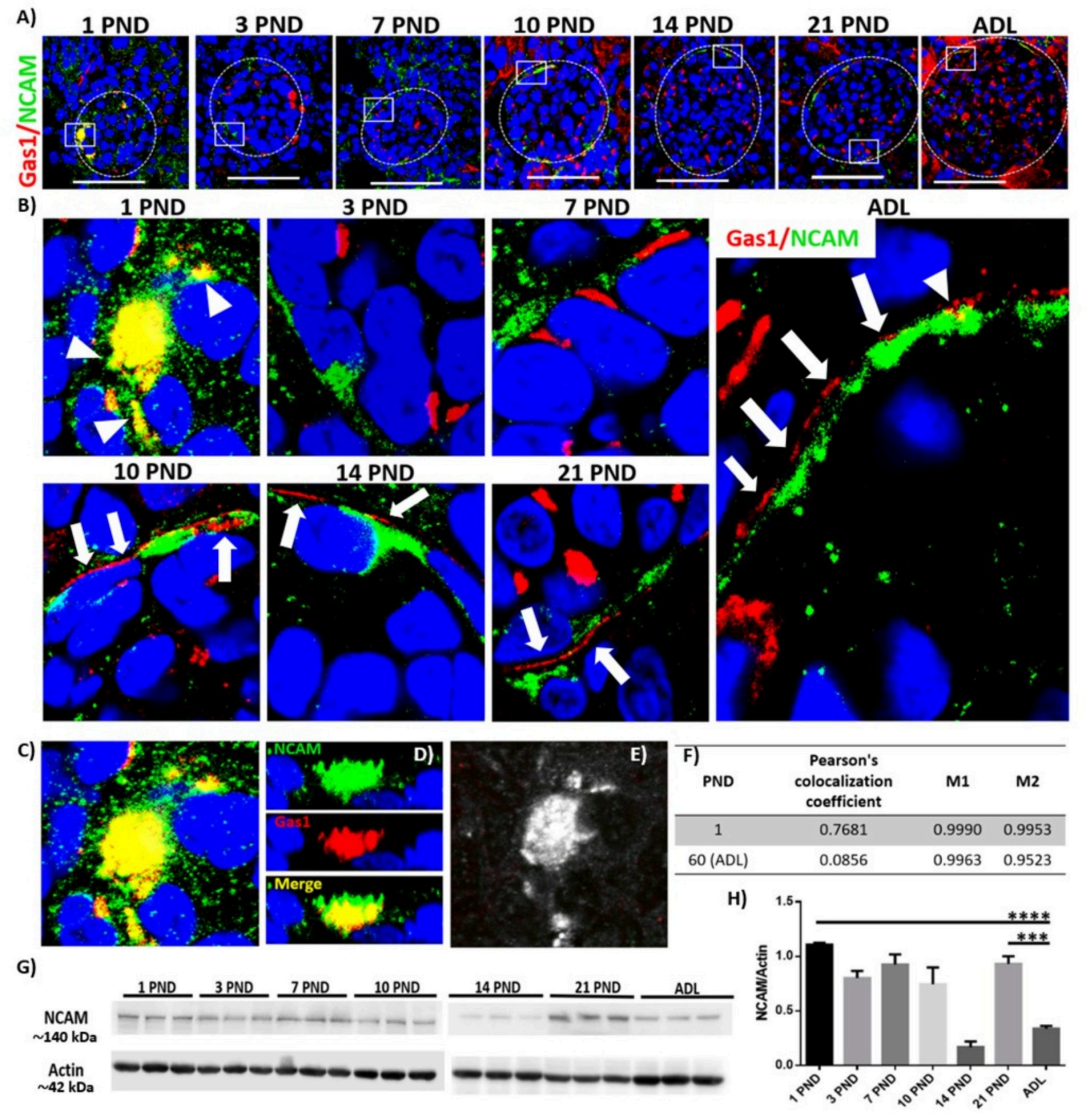
Gas1 and NCAM in the glomerulus and Bowman’s capsule during postnatal renal development and in adulthood of the rat. A) Immunofluorescence of the general expression pattern of Gas1 and NCAM in the glomerulus during the different postnatal days (PND) evaluated. NCAM is expressed in the glomerulus and Bowman’s capsule (BC) and co-localizes with Gas1. B) magnification of the boxes in A, NCAM is expressed intraglomerularly to PND 1 and colocalized with Gas1 (arrowhead), as the kidney matures, colocalization decreases, but they remain very close to each other in the BC (white arrows). Two types of analysis were made to confirm the colocalization of Gas1 and NCAM to PND 1 (C), the first was qualitative with a Z-stacks of the colocalization zone (D); and the second quantitative, by calculating the Pearson colocalization coefficient (PND 1 r = 0.7681, ADL r = 0.0856, F), that generates a colocalization map (E, the areas where the two proteins colocalize in PND 1 are seen in white) and the calculation of the Manders coefficient (PND 1 M1 = 0.9990 and M2 = 0.9953, ADL M1 = 0.9963 and M2 = 0.9523). G and H) analysis of NCAM expression by Western blot in an enriched sample of glomeruli, we find that the highest expression is in PND 1, 7 and 21, and the lowest expression is in PND 14 and ADL. Each PND analyzed is expressed as the relative density of a group of 20 (younger ages) and 8 (21 PND and adult) rats normalized with actin as loading control. Gas1 (red), NCAM (green), Merge (yellow) and nuclei with DAPI (blue) are shown. The glomerulus is delimited by a white circular line. The white squares represent the magnification of specific areas in the glomerulus. Scale bars = 50 μm. Values are means ± standard deviation (SD). One-way ANOVA. *p<0.05, **** p<0.0001. Immunofluorescence experiments were performed in kidney sections. A representative experiment is shown (n = 3).

There is evidence of a subpopulation of progenitor cells present in Bowman’s capsule in the adult kidney and it has been suggested that Gas1 could be maintaining this population of cells in a quiescent state [13, 36]. To elucidate whether there is a relationship between Gas1 and RPC, we used NCAM as a specific marker of progenitor cells. Regarding the expression of Gas1, we observed the same expression pattern described previously (Fig 2). Interestingly we found that NCAM is also expressed intraglomerularly at PND 1 (Fig 3A), that both proteins (Gas1/NCAM) strongly colocalize in the first PND (Fig 3B, arrowheads, C and E) and that NCAM is present throughout postnatal development and maintains its homogeneous expression in Bowman’s capsule in the adulthood. The colocalization of Gas1 and NCAM was verified with a *Z-stacks* (Fig 3D) and with the Pearson colocalization coefficient analysis (see methods) we confirmed that both proteins colocalize strongly (r = 0.7681, Fig 3F). Interestingly, after PND 1 Gas1 only co-localizes with NCAM in very restricted areas during postnatal development and in adults (Fig 3B ADL, arrowhead) as observed in the result of the Pearson coefficient of ADL (r = 0.0856), the value of r is very low compared to that of PND 1. It seems that as the kidney matures there is a spatial rearrangement of both proteins since their colocalization decreases, but however, they remain very close to each other in the BC (Fig 3B, white arrows), as determined by the coefficient analysis of Manders (PND 1 M1 = 0.9990 and M2 = 0.9953; ADL M1 = 0.9963 and M2 = 0.9523) where we observe very similar values in both stages of development confirming that the expression of both Gas1 and NCAM maintain a close spatial relationship. We also found that NCAM has an inverse expression pattern compared to Gas1, that is, while the highest level of expression of Gas1 in the glomerulus is in the adult kidney (Fig 2E), the highest level of NCAM expression is on the first day after the rat is born and it decreases significantly in the adulthood, as determined by Western blot analysis (Fig 3G and 3H).

### Gas1 is expressed in the cytoplasm of the proximal convoluted tubule in the kidney during postnatal development and increases its expression as the kidney matures

**Fig 4:**
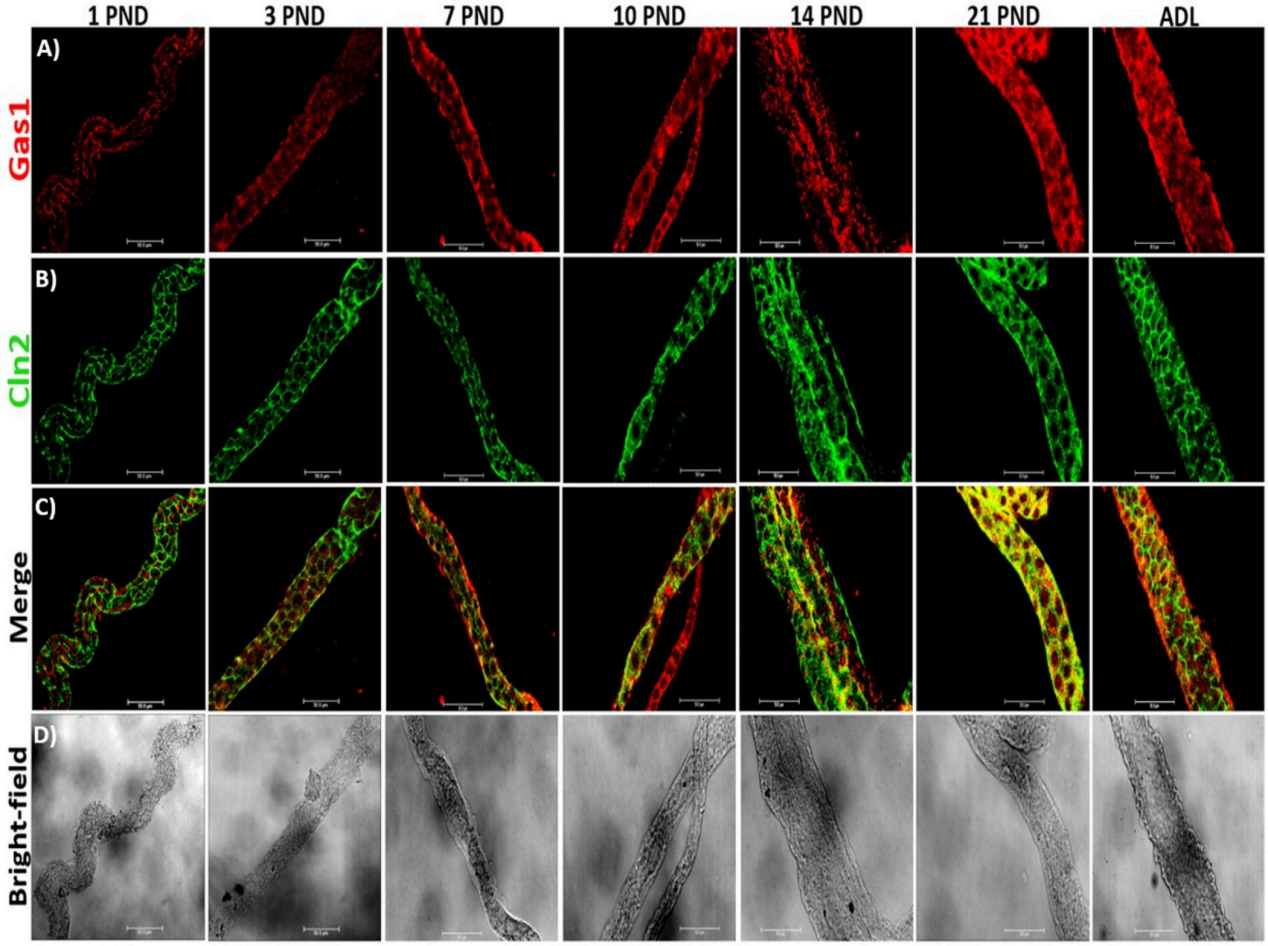
Gas1 is expressed in the proximal convoluted tubule in the kidney during postnatal development and in adulthood of the rat. A) immunofluorescence analysis of the general expression pattern of Gas1 in proximal tubules isolated from the renal cortex at different postnatal ages (PND) studied. We found that Gas1 is expressed cytoplasmically and increases as the kidney matures. B) Claudin protein 2 (Cln2) as a specific marker of the proximal region of the nephron. C) Merge of the Gas1 and Cln2 proteins, it can be observed that Gas1 co-localizes with Cln2 mainly at PNDs 14 and 21. D) bright-field, the classic proximal tubular contoured morphology is observed. In PND 14 and 21 there is more than one proximal tubule. In PND 10 there is a proximal tubule (Cln2+) and another tubule that is not proximal (Cln2-). Gas1 (red) and Cln2 (green) are shown. Scale bars = 50 μm. Immunofluorescence experiments were performed in isolated renal tubules. A representative experiment is shown (n = 3).

**Fig 5:**
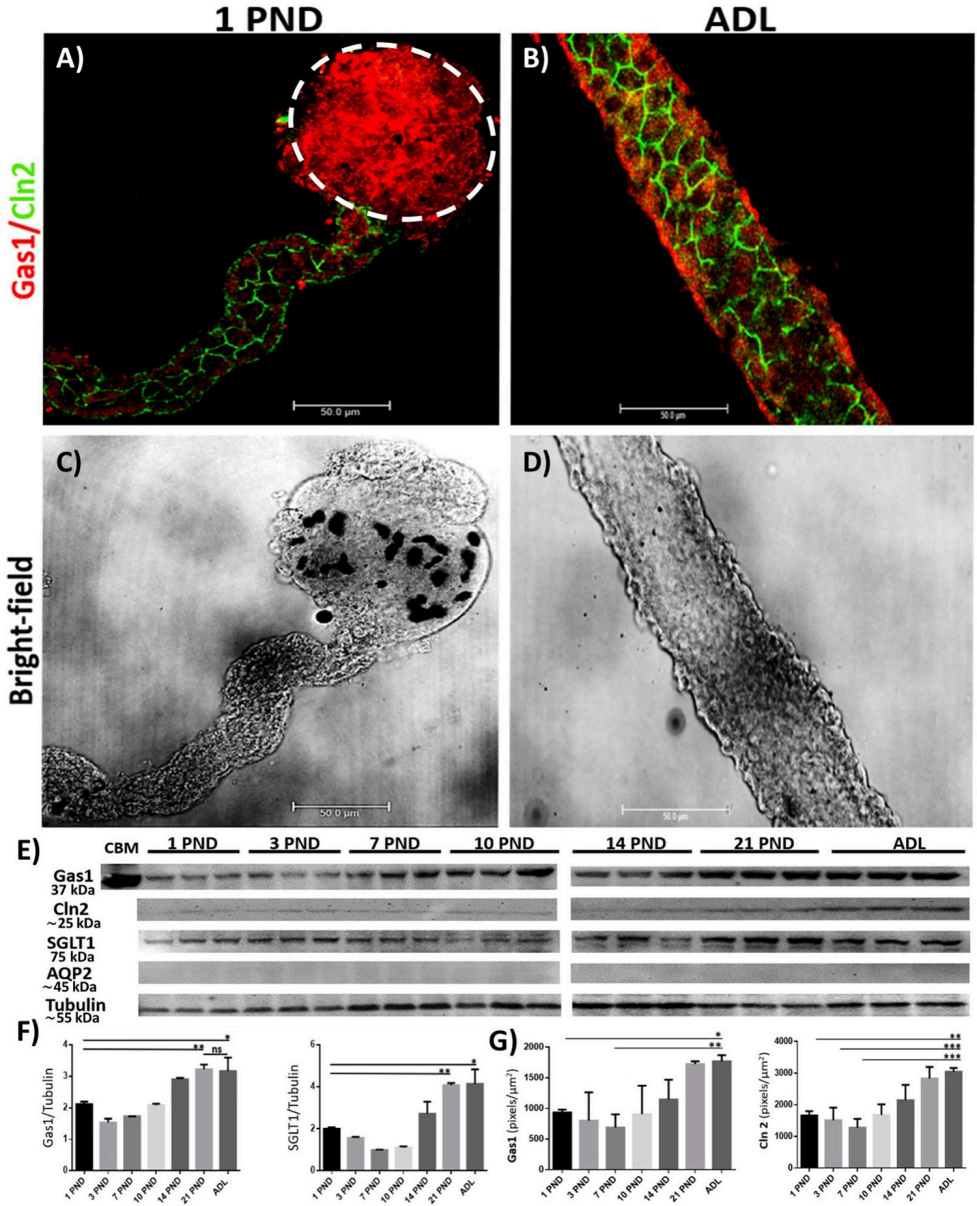
Comparison of Gas1 expression and claudin 2 (Cln2) in the proximal convoluted tubule in the newborn and adult rat. A and B) immunofluorescence of proximal convoluted tubules isolated from the renal cortex of rats from PND 1 and ADL. We found a pattern of increasing expression of Gas1 and Cln2 as the kidney matures. C) bright-field of an isolated proximal tubule attached to its respective glomerulus from PND 1. D) bright-field of an isolated proximal tubule from an adult rat, found to be thicker compared to that of 1 PND. The immunofluorescence methodology used for the tubules and the glomerulus are different, for this reason the signal observed from Gas1 in the glomerulus (A, delimited by a white circular line) does not correspond to the expression described previously (Fig 2), the image is shown as a positive control of the proximal tubule, since anatomically only the proximal tubule is attached to the glomerulus. E) Western blot analysis of Gas1 in an enriched sample of proximal tubules, we used as a negative control the protein (distal nephron protein) aquaporin 2 (AQP2). As a positive control for Gas1 we used a sample of the cerebellum (CBM). We did not find a significant signal from AQP2, confirming that the analyzed samples were mainly from the proximal region. F) densitometric analysis of the Western blot of Gas1 and SGLT1. Each PND analyzed is expressed as the relative density of a group of 20 (younger ages) and 8 (21 PND and adult) rats normalized with α-tubulin as loading control. G) semi-quantitative analysis of Gas1 and Cln2 in the proximal tubule. Gas1 (red) and Cln2 (green) are shown. Scale bars = 50 μm. Values are means ± standard deviation (SD). One-way ANOVA. *p<0.05, **** p<0.0001. Immunofluorescence experiments were performed in isolated renal tubules. A representative experiment is shown (n = 3).

Since it has been previously reported that Gas1 exerts different functions depending on the cell/tissue context, we decided to explore whether Gas1 was expressed in the proximal convoluted tubule. Previously, we had not observed the presence of Gas1 in the membranes of epithelial cells in the proximal tubules [13, 37] but now decided to explore whether it was found in the cytosol of these cells. To achieve this objective, we performed a deeper permeabilization of the tissue [38–40], to improve antibody penetration and immunofluorescence staining intensity in proximal tubule epithelial cells, as described in the Materials and methods section. Employing these strict conditions, we could observe that Gas1 is expressed in the cytosol of the epithelial cells of the proximal tubule although at a low level. Under the same conditions we also detected the co-localization of Gas1 and Cln2 in postnatal days 10, 14 and 21 (Fig 4). Immunofluorescence studies of proximal tubules isolated from the renal cortex at the different postnatal ages studied, indicated that Gas1 is present since birth and that its expression increases as the kidney matures (Fig 4A). As a positive control of this region of the nephron we used the tight junction protein claudin 2 (Cln2) [41, 42], we can observe that the characteristic "chicken fence" expression pattern of Cln2 is present from PND 1, interestingly we found that its expression also increases as the kidney matures and in PND 21 there is more expression of Gas1 in the cytoplasmic. Moreover, Cln2 co-localizes with Gas1, and we observed that Gas1 is expressed in the cytoplasm from PND 1 and increases its expression as the kidney matures. This significant increase in the expression of Gas1 and Cln2 is best observed when we compare the immunofluorescence of PND 1 with ADL (Fig 5A and 5B), we also observed an evident increase in the size of the proximal convoluted tubule in the adult (Fig 5C and 5D). By Western blot analysis of an enriched sample of proximal tubules, we confirmed that there is a statistically significant progressive increase in the expression of Gas1, Cln2 and SGLT1 (Sodium Glucose Co-transporter-1, used as another positive control for the proximal tubule, Fig 5E), related to the maturation of the proximal tubule, since the highest expression of these proteins is found in the adult (Fig 5F, Cln2 analysis is not shown). Moreover, similar results were obtained using a semi-quantitative analysis of Gas1 and Cln2 expression since both proteins show statistically significant increases as the kidney matures (Fig 5G).

### Renal progenitor cells are found throughout postnatal development in the proximal convoluted tubule and decreases in adult rats

**Fig 6:**
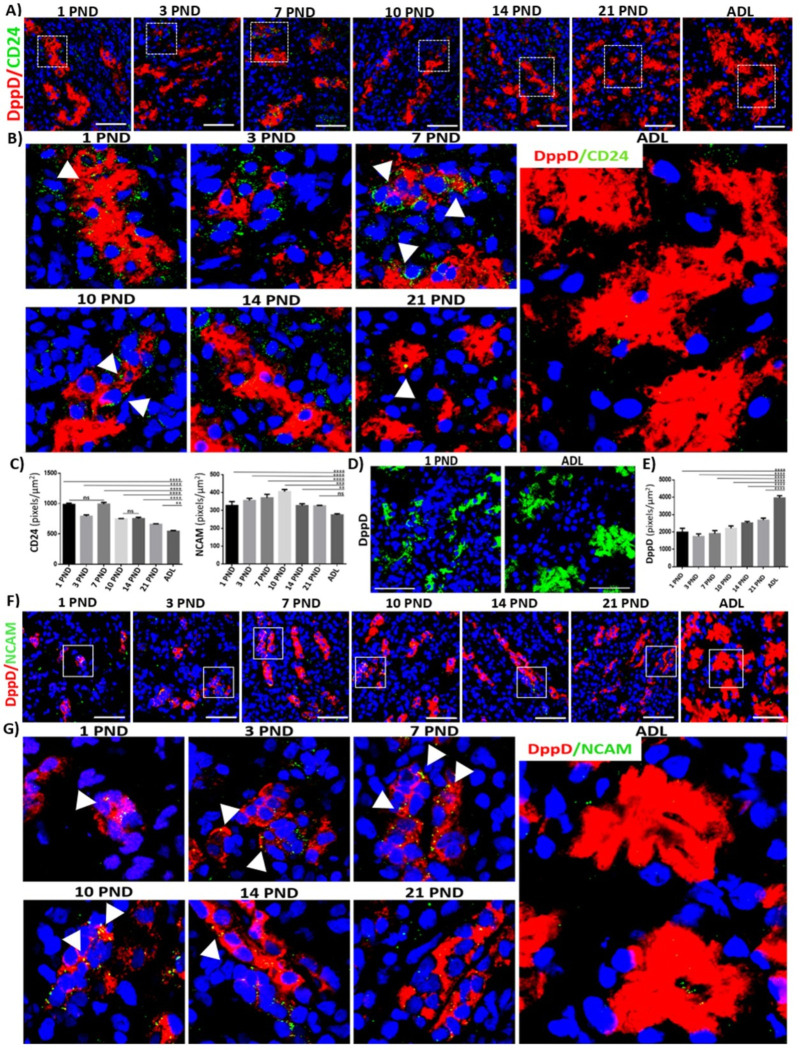
Expression of renal progenitor cell (RPC) markers in the proximal convoluted tubule during postnatal development and in adulthood of the rat. Immunofluorescence analysis of the general expression pattern of two RPC markers, CD24 (A) and NCAM (F) during postnatal renal maturation. B and G) magnification of the boxes in A and F, respectively, there is a higher expression of RPC markers in the proximal convoluted tubules of newborn rats and they decrease significantly in the adult, this is reflected in their semi-quantitative analysis (C). Small areas are observed where RPCs markers colocalize with DppD (arrowhead). We found a significant increase in the expression of DppD when comparing PND 1 with the adult (D, DppD in green), consistent with the semi-quantitative analysis (E). DppD (red A, B, F, G), CD24 (green, A and B) NCAM (green, F and G), Merge (yellow) and nuclei with DAPI (blue) are shown. The white squares represent the magnification of specific areas in the proximal convoluted tubule. Scale bars = 50 μm. Values are means ± standard deviation (SD). One-way ANOVA. *p<0.05, **** p<0.0001. ns = not significant. Immunofluorescence experiments were performed in kidney sections. A representative experiment is shown (n = 3).

Based on our observations of the glomerulus, we decided to determine the pattern of expression of markers of renal progenitor cells (RPC) in the proximal convoluted tubule during postnatal development. Using immunofluorescence, we analyzed the expression of CD24 and NCAM, both RPC markers, finding that both proteins show a similar pattern of expression during kidney development. We used the brush border protein dipeptidyl-peptidase (DppD) as a positive control for this region [28] and observed that DppD increases significantly as the proximal tubules mature (Fig 6D and 6E). We observed that CD24 is expressed from PND 1 in the proximal convoluted tubule and colocalizes in restricted areas with DppD mainly in the first postnatal days, then its expression decreases significantly as the proximal tubules mature (Fig 6A and 6B, arrowhead) reaching the lowest level of expression in the adult proximal tubules (Fig 6C). Similar results were observed with NCAM, we observed that NCAM is expressed from PND 1, and colocalizes with DppD in limited regions during postnatal development (Fig 6F and 6G, arrowhead) and that the expression of NCAM also decreases significantly as the proximal tubules mature. (Fig 6C). With these results, we conclude that the general expression pattern of RPCs in the proximal convoluted tubule is higher in newborn rats and decreases significantly as the tubules mature.

### Gas1 is expressed in some tubules in the distal nephron during postnatal development, the expression of Gas1 becomes homogeneous and stable in the distal region until adulthood in the rat

**Fig 7:**
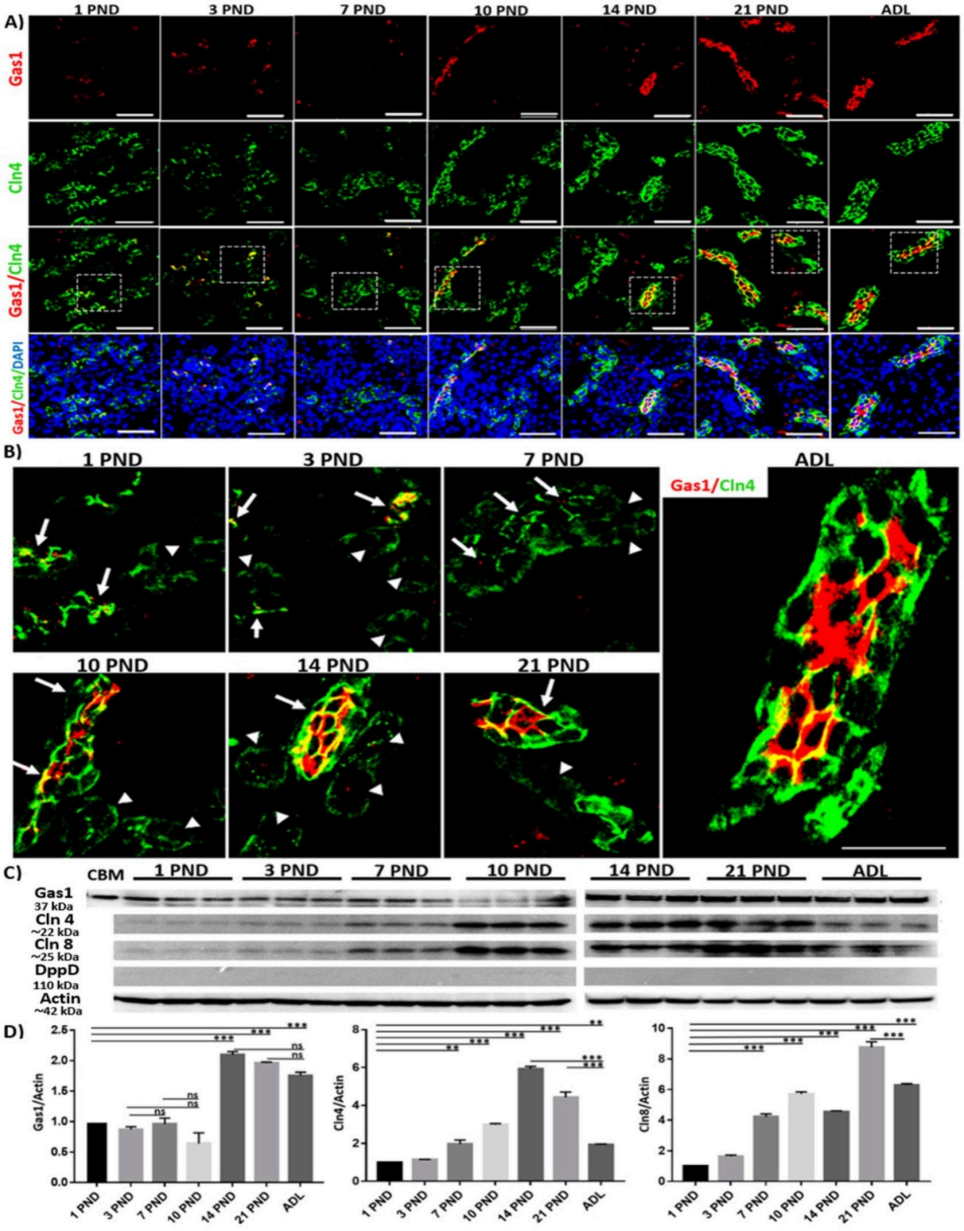
Gas1 expression in the distal nephron during postnatal development and in adulthood of the rat. A) immunofluorescence analysis of the general expression pattern of Gas1 in the distal nephron on the different postnatal days (PND) studied, we found that Gas1 is expressed in the cell membrane and its expression is homogeneous and constant in the adult distal tubule. B) magnification of the boxes in A, Gas1 is present in some distal tubules (white arrow) but not in others (arrowhead) and the expression of Gas1 is homogeneous and constant only in the adult stage. C) Western blot analysis of Gas1 in an enriched sample of distal tubules. To ensure that the results obtained were specifically from the distal region, we used the DppD protein (typical of the proximal tubules) as a negative control. As a positive control for the Gas1 protein, we used a sample of the cerebellum (CBM). We did not find a significant DppD signal, confirming that the analyzed samples were mainly from the distal region. We found that Gas1, Cln4 and Cln8 are expressed from the first day the rat was born and increase their expression as the kidney matures. D) densitometric analysis of the Western blot of Gas1, Cln4 and Cln8. Each PND analyzed is expressed as the relative density of a group of 20 (younger ages) and 8 (21 PND and adult) rats normalized with actin as loading control. Gas1 (red) and Cln4 (green) and nuclei with DAPI (blue) are shown. The white squares represent the magnification of specific areas in the distal tubule. Scale bars = 50 μm. Values are means ± standard deviation (SD). One-way ANOVA. *p<0.05, **** p<0.0001. ns = not significant. Immunofluorescence experiments were performed in kidney sections. A representative experiment is shown (n = 3).

Since Gas1 is expressed in the glomerulus, Bowman’s capsule, and the proximal tubule, we explored its pattern of expression in the distal nephron during postnatal development. To identify this region of the nephron we used the tight junction proteins claudin 4 (Cln4) and claudin 8 (Cln8) as specific markers of this region [41, 43]. Using immunofluorescence, we found that Gas1 is expressed in the distal nephron from day one after birth, throughout postnatal development and significantly increases its the expression in the adult. We found that Gas1 colocalizes with Cln4 which is expressed mainly in the cell membrane of the distal tubules and increases its expression as the kidney matures (Fig 7A). Interestingly, we found that Gas1 is not expressed in all distal tubules during the first postnatal days, that is, some distal tubules are expressing Gas1, and others do not seem to express Gas1 (Fig 7B, white arrow, and arrowhead, respectively), but after PND 21 Gas1 was homogeneously expressed in all tubules of the distal nephron and remained in the adult distal tubules (Fig 7B ADL). The above is very interesting because, since we found that Gas1 is expressed in all tubular structures of the distal nephron, maintaining a permanent and homogeneous expression in the adult stage, we suggest a possible role for Gas1 as a marker of the state of cell differentiation in the distal nephron. We confirmed by Western blot analysis in an enriched sample of distal tubules the expression pattern of Gas1 described above; Gas1 is expressed from PND 1 and the expression increases significantly in the adult stage. We also observed a very similar pattern of expression between claudins 4 and 8, with a low expression level in PND 1 and increasing its expression as the kidney matures (Fig 7C and 7D). Interestingly, Gas1 has an expression pattern similar to the one described in the glomerulus and proximal tubules, that is to increase its expression as the kidney matures.

### Gas1 is expressed in renal progenitor cells of the distal nephron and has an inverse expression pattern during postnatal development and in the adult rat

**Fig 8:**
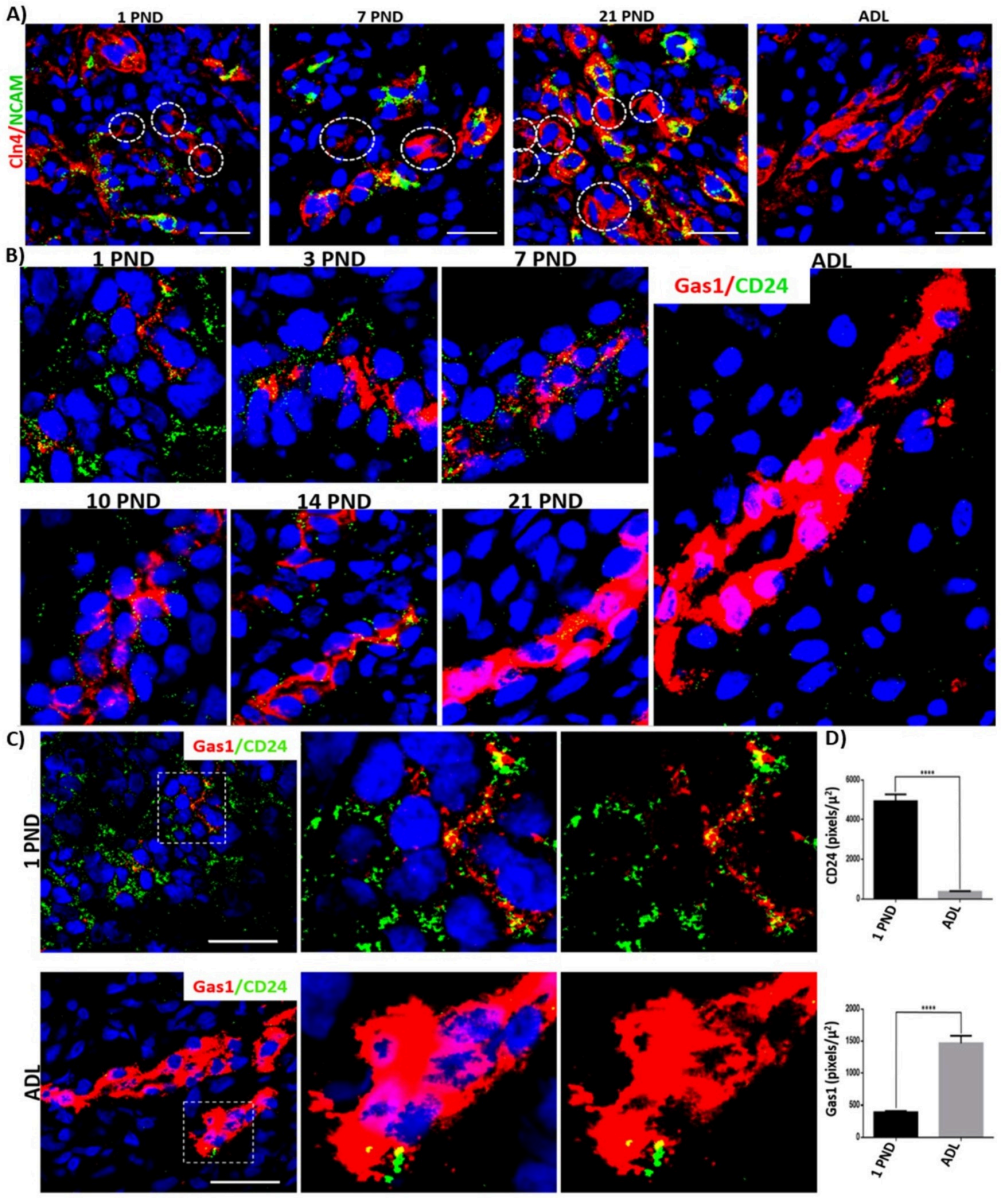
Expression of renal progenitor cell (RPC) markers in the distal nephron during postnatal development and in adulthood of the rat. A) immunofluorescence analysis of the general expression pattern of NCAM in the distal tubules, we found that Cln4 (red) and NCAM (green) are expressed from PND 1, that both proteins colocalize and that NCAM is not expressed in all distal tubules during kidney maturation (dotted circles). B) CD24 (green), is expressed from PND 1 and colocalizes with Gas1 (red) in different areas of the distal tubules during postnatal development, and that the expression of CD24 decreases in the adult nephron. C) Comparison of Gas1/CD24 expression in PND 1 and ADL. D) Semi-quantitative analysis of the comparison of Gas1/CD24 expression in PND 1 and ADL. The white squares represent the magnification of specific areas in the distal tubule. Cln4 (red, A), NCAM (green, A), Gas1 (red, B and C), CD24 (green, B and C), Merge (yellow), and nuclei with DAPI (blue) are shown. Scale bars = 25 μm. Values are means ± standard deviation (SD). *p<0.05, **** p<0.0001. Immunofluorescence experiments were performed in kidney sections. A representative experiment is shown (n = 3).

Employing immunofluorescence, we evaluated the expression pattern of renal progenitor cells (RPC) in the distal nephron during postnatal development of the rat. We found that the expression pattern of RPCs (identified by the presence of NCAM+/CD24+) is higher in the distal tubules of newborn rats and significantly decreases in the adult distal tubules. Thus, we confirm the presence of RPCs in the distal nephron during development and their significant decrease in the adult kidney (Fig 8A). Interestingly, we noticed that NCAM is not present in all tubules of the distal nephron during renal maturation (Fig 8A, dotted circles). We wanted to assess whether Gas1 was expressed in the RPCs of the distal nephron since a population of cells with stem cell characteristics has been previously reported in the distal nephron [44]. We found that indeed, Gas1 is expressed in the RPCs of the distal nephron during the postnatal renal maturation process, since it colocalizes with CD24 from PND 1 and throughout postnatal development (Fig 8B). A very interesting observation was that Gas1 and CD24 have inverse expression patterns (Fig 8C and 8D), that is, the highest expression level of CD24 is observed in PND 1 and the lowest expression level is observed in PND 21 (the signal decreases significantly in ADL) (Fig 8B and 8D), while for Gas1 it is the opposite, the lowest levels of its expression are found in the first postnatal days and increase during the process of renal maturation, having the highest level of expression in adults (Fig 7C).

## Discussion

Gas1 is a pleiotropic protein, thus its function(s) depend on the stage of development, cycle and/or cellular context in which it is expressed [22]. Since the presence of Gas1 has a fundamental role in the preservation of renal progenitor cells during embryonic development [15], we considered relevant to examine whether Gas1 was expressed in the different regions of the nephron and, if so, whether there was any relationship between Gas1 and RPCs during postnatal renal maturation, since RPCs remain active during postnatal nephrogenesis and are conserved in the adult kidney.

In summary, we found that Gas1 is expressed in various regions of the nephron (Bowman’s capsule, glomerulus, proximal convoluted tubule, and distal nephron) and that it has a dynamic expression pattern as the kidney matures. Moreover, we found that there is an inverse pattern of expression between Gas1 and RPCs; while Gas1 increases as the kidney matures, the expression of RPC markers decreases significantly.

### Increased kidney function and decreased proteinuria related to kidney maturation

We observed a decreased glomerular filtration in the first postnatal days that was reversed as the kidney matured and a high proteinuria in the newborn rat that decreased significantly in the adult. We also determined that glomerular filtration increased significantly after PND 14, these results are consistent with that observed by Neiss and Klehn [16], who found that renal morphogenesis is not complete until PND 15. It is known that during the first months of life, renal function parameters are quantitatively lower than those of adults [19, 45, 46]. The maturity of the renal structures is reached when the renal function parameters are achieved, such as the elevated glomerular filtration rate and the decreased proteinuria characteristic of adults [18]. These renal function parameters remain stable in the 8-week-old rat, reflecting that the renal structures have reached full maturity [16]. With the above functional results, we demonstrated an appropriate model of renal maturation for this study.

### Gas1 expression in the nephron during the postnatal renal maturation process and in adulthood

We will discuss the main findings of this work following an anatomical order in the nephron:

*Bowman’s capsule*: we found that Gas1 is expressed from PND 10 on and maintains its expression in the adult, because the expression of Gas1 is homogeneous and constant in the adult. We suggest Gas1 as a possible marker of the state of differentiation of this kidney structure. Another novel finding was that Gas1 and NCAM have an inverse expression pattern during renal maturation and in adults. In our experimental model with healthy rats undergoing a normal process of renal maturation, we assumed that the RPCs that are active from PND 1 (nephrogenesis ends in the first postnatal days) [14] start to differentiate and therefore throughout maturation these markers decrease significantly. It has been previously described that NCAM is also expressed in the early epithelialization stages [47], perhaps it is for this reason that we observed a significant decrease in NCAM expression from postnatal day 21 up to the adult. Our results suggest that since Gas1 is homogeneously expressed in Bowman’s capsule (like NCAM), and because Gas1 is expressed in the RPCs from PND 1 and reaches its highest expression in the adult, and then remains constant, it could negatively regulate RPCs in Bowman’s capsule during postnatal development and in adulthood, because the expression pattern of Gas1 is inverse to the pattern observed for NCAM, and also because the decrease in the expression of Gas1 allows the activation of the RPCs in glomeruli in a model with adult rats with damage due to hyperglycemia [13]. Whether or not Gas1 is capable of interacting with NCAM or a homologue remains an open question [48].

*Glomerulus*: we found that Gas1 is expressed in the glomerulus from PND 1, increasing its expression in the adult and that it is not expressed in the slit diaphragm at any stage of postnatal development or in the adult. Probably the intraglomerular expression that we found of Gas1 from PND 1 and that is maintained in the juvenile and adult rat may be related to the expression in mesangial cells and podocytes as previously described by van Roeyen [12] in adult rats. Interestingly, we observed two bands for Gas1 in the Western Blot analysis. We had previously reported that there are two isoforms of Gas1 (one of 37 kDa and the other ≈ 34 kDa) [49]. Perhaps the second band found in the glomerulus is another Gas1 isoform weighing <37 kDa previously described. Thus, it will be relevant to identify this isoform of Gas1 found from the beginning of postnatal development in the glomerulus to better understand its signaling properties, because another group has reported that, the endogenous form of Gas1 they described induced cell cycle arrest in mesangial cells [12]. Evidently Gas1 is important during renal maturation (since it is present since the rat is born, its expression increases and it maintains its highest level in the adult) and since we have observed this pattern of expression in the glomerulus, in contrast with our findings in the proximal and distal tubules, where we only detect one Gas1 isoform, it is likely that a different function and regulation of Gas1 occurs in different regions of the nephron. Future studies will be necessary to unmask these functional differences, during the postnatal renal maturation.

*Proximal convoluted tubule*: we found that Gas1 is expressed in the cytoplasm of epithelial cells of the proximal tubule from PND 1, that its expression increases as the kidney matures, and that Gas1 expression is maintained in fully differentiated adult cells. To obtain these results, we had to modify the immunofluorescence protocol we had previously employed [13, 37] (see Experimental procedures) [38–40, 50, 51] to determine Gas1 expression in other regions of the cell (besides the cell membrane). These data highlight that the penetration of the antibody and the intensity of the immunofluorescence staining in the epithelial cells of the proximal tubule are critical. Therefore, the protocols used for immunofluorescence for glomerulus, proximal tubules, and distal tubules are all different. Considering that Gas1 is a GPI-anchored protein and that a common characteristic of this type of proteins is that they are found both membrane-bound and in soluble forms, our results suggest that in the proximal tubule a soluble isoform may be present, since we found Gas1 in the cytosol of these cells. This is a very relevant observation since the proximal convoluted tubule is the region of the nephron that performs almost all the reabsorption and secretion of the main solutes in the kidney [52]. And since there is evidence indicating that Gas1 is secreted by podocytes and/or mesangial cells in the adult [12] and by perivascular cells in the liver [49], we believe that more work is necessary in the future to determine the specific cell populations of the nephron that express Gas1 and can also secrete it. We hypothesize that Gas1 may participate in local signaling (autocrine and/or endocrine), as well as paracrine communication and perhaps at as of yet scarcely explored systemic level, since a wide distribution of the soluble form of Gas1 has been found in the body fluids of the rat (cerebrospinal fluid (CSF), blood plasma [49] and urine [12]). Interestingly, in a previous report, no significant difference was found between the levels of soluble Gas1 present in blood plasma and CSF [49], reflecting the important role that the kidney may play in regulating the concentration of Gas1 in order to maintain isotonic concentration.

On the other hand, the proximal convoluted tubule: a) has the highest rate of cell proliferation in the healthy kidney (it is particularly susceptible to tubular damage/necrosis) [53]; b) the S3 segment has the ability to proliferate throughout life and c) its rapid ability to recruit cells to enter division and proliferate is due to the fact that it has a large pool of cells arrested in the G1 phase [54]. Thus, we considered that perhaps this section of the nephron could have different cell cycle controls in G1-S progression compared to other tubular structures, and therefore it is likely that Gas1 is participating as another control arresting the cell cycle of fully differentiated cells that remain quiescent after they reach "maturity". Our findings are consistent with a possible role of Gas1 as a potential cell cycle regulator in the proximal convoluted tubule. We believe this to be the case, because we found that Gas1 increases significantly as the kidney matures, and because although kidney morphology is completely developed from DNP 15 [16], the nephron continues its longitudinal growth until reaching its final development and therefore, the epithelial cells continue to divide until reaching "renal maturity" [21]; this is confirmed by the increase in tubular size.

*Distal nephron*: we observed Gas1 from PND 1, but not in all distal tubular structures during renal postnatal development (as we found in the proximal tubules), this led us to consider whether there are different patterns of postnatal maturation between tubular structures in the nephron. And if so, does this difference between their proximal and distal postnatal maturation patterns depend on their biological function? Interestingly, the expression of Gas1 is homogeneous and constant just after DPN 21 when all the distal structures have already matured [16], for this reason we suggest that Gas1 could be a potential marker of the state of cell differentiation at the level of the distal nephron.

### Renal progenitor cells in the proximal and distal tubule

The significant decrease of RPCs that we observed in adults is due to the differentiation of tubular epithelial cells and therefore the consequent loss of markers that are expressed in nephron progenitor cells during nephrogenesis and which are downregulated in fully differentiated cells [55], it has also been reported that NCAM is expressed in the first stages of epithelialization [47]. We also found that Gas1 and RPC markers have an inverse pattern of expression during postnatal renal development, this result is important since it could indicate a role of Gas1 as a negative regulator of RPCs in the proximal and distal structures during renal maturation and in adulthood, in a manner similar to that observed in Bowman’s capsule. The possible role of Gas1 as a regulator of progenitor cells has been previously described in the postnatal brain, and it has been suggested that Gas1 could be regulating cell cycle in neural cells; it is also known that Gas1 is expressed in stem cells that are in the G0 phase unless they are activated and enter the cell cycle [5].

Specific markers of each region of the nephron; Nephrin, Cln2/SGLT1/DppD and Cln4/Cln8 were used to identify the glomerulus, proximal tubule, and distal nephron, respectively. We found that these markers increase as the kidney matures, these results reflect the tubular and glomerular structural immaturity at the time of birth and the process of cellular maturation that the kidney undergoes during the postnatal period [56, 57].

We consider that more studies are needed to fully understand the function of Gas1 during renal maturation, since the objective of the present work was only to characterize its expression pattern throughout the nephron due to the scarcity/lack of information about Gas1 and postnatal development, however, our results suggest a potential role for Gas1 in this critical stage of kidney development in the rat.

In conclusion, the finding that Gas1 is present and significantly increases its expression during the postnatal maturation process in the kidney, suggests that Gas1 could be related to the control of the cell cycle [2], probably maintaining the arrested cell cycle of completely differentiated cells in the nephron.

## Supporting information

S1 Raw images (PDF)
