# Supplementary material for: Characterization of the pattern of expression of Gas1 in the kidney during postnatal development in the rat
Source: PLoS One. 2023 Apr 24;18(4):e0284816. doi: 10.1371/journal.pone.0284816 (PMC10124827; doi:10.1371/journal.pone.0284816)

All western blots were detected in an EC3 imaging System (UVP, BioImaging Systems).

Figure 2 and 3. Western blot of an enriched sample of glomeruli. Each PND analyzed is expressed as the relative density of a group of 20 (PND 1, 3, 7, 10 and 14) and 8 (21 PND and adult) rats normalized with actin as loading control.

Figure 2 D. Western blot of an enriched sample of glomeruli.

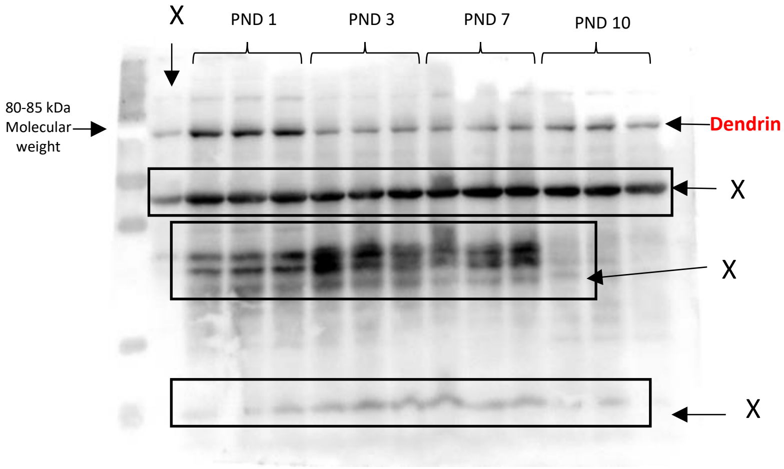

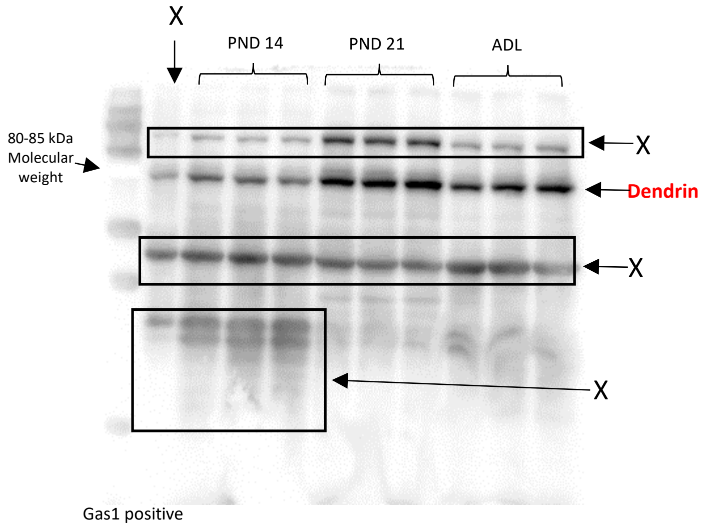

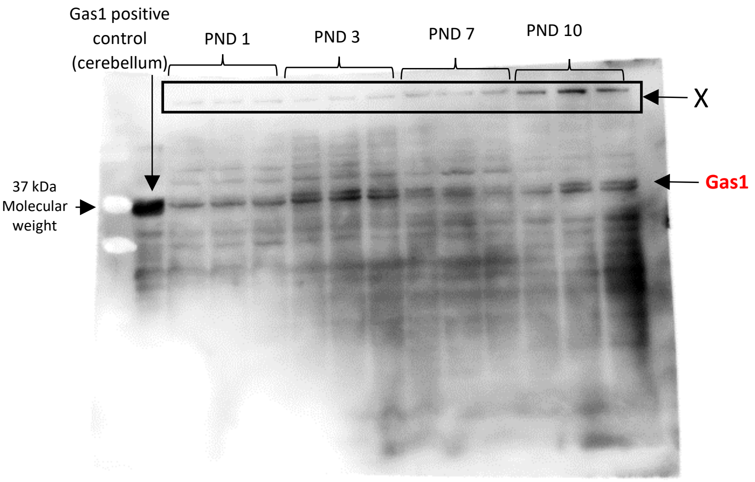

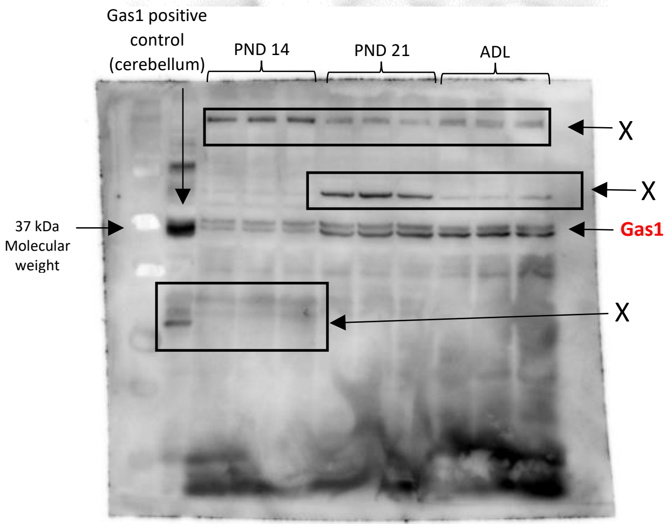

Figure 2 D and 3 G. Western blot of an enriched sample of glomeruli.

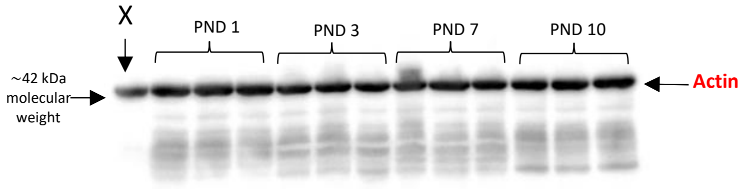

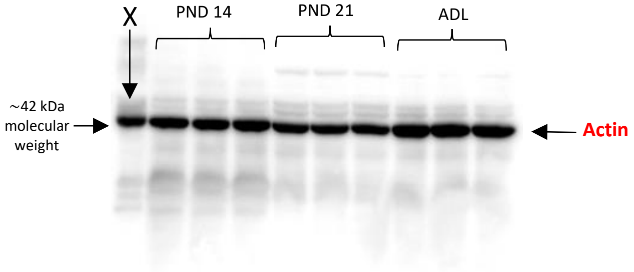

Figure 3 G. Western blot of an enriched sample of glomeruli.

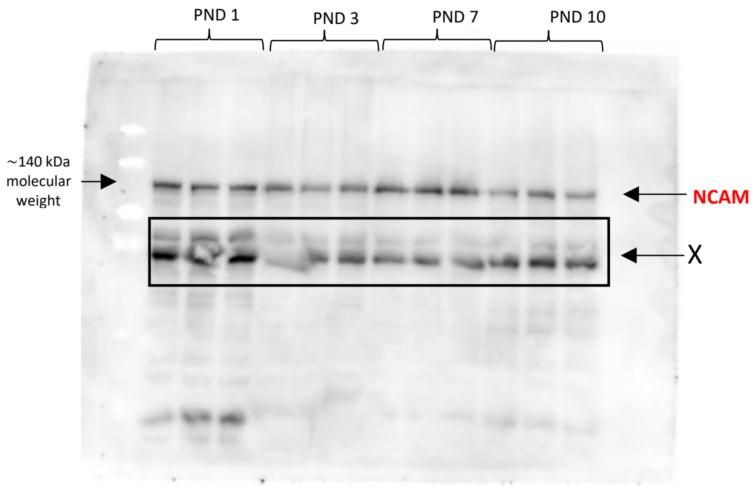

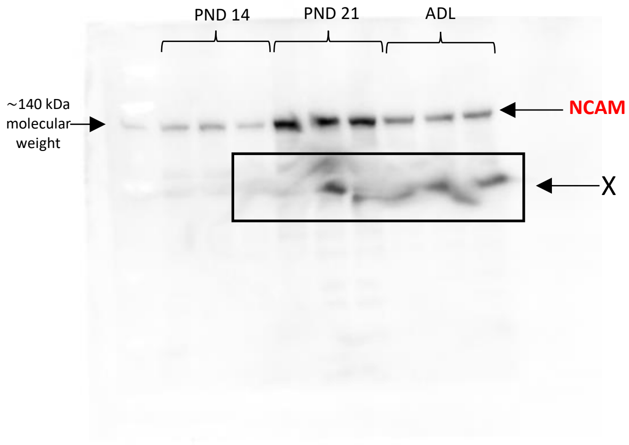

Figure 5 E. **Western blot of an enriched sample of proximal tubules. Each PND analyzed is expressed as the relative density of a group of 20 (PND 1, 3, 7, 10 and 14) and 8 (21 PND and adult) rats normalized with tubulin as loading control.**

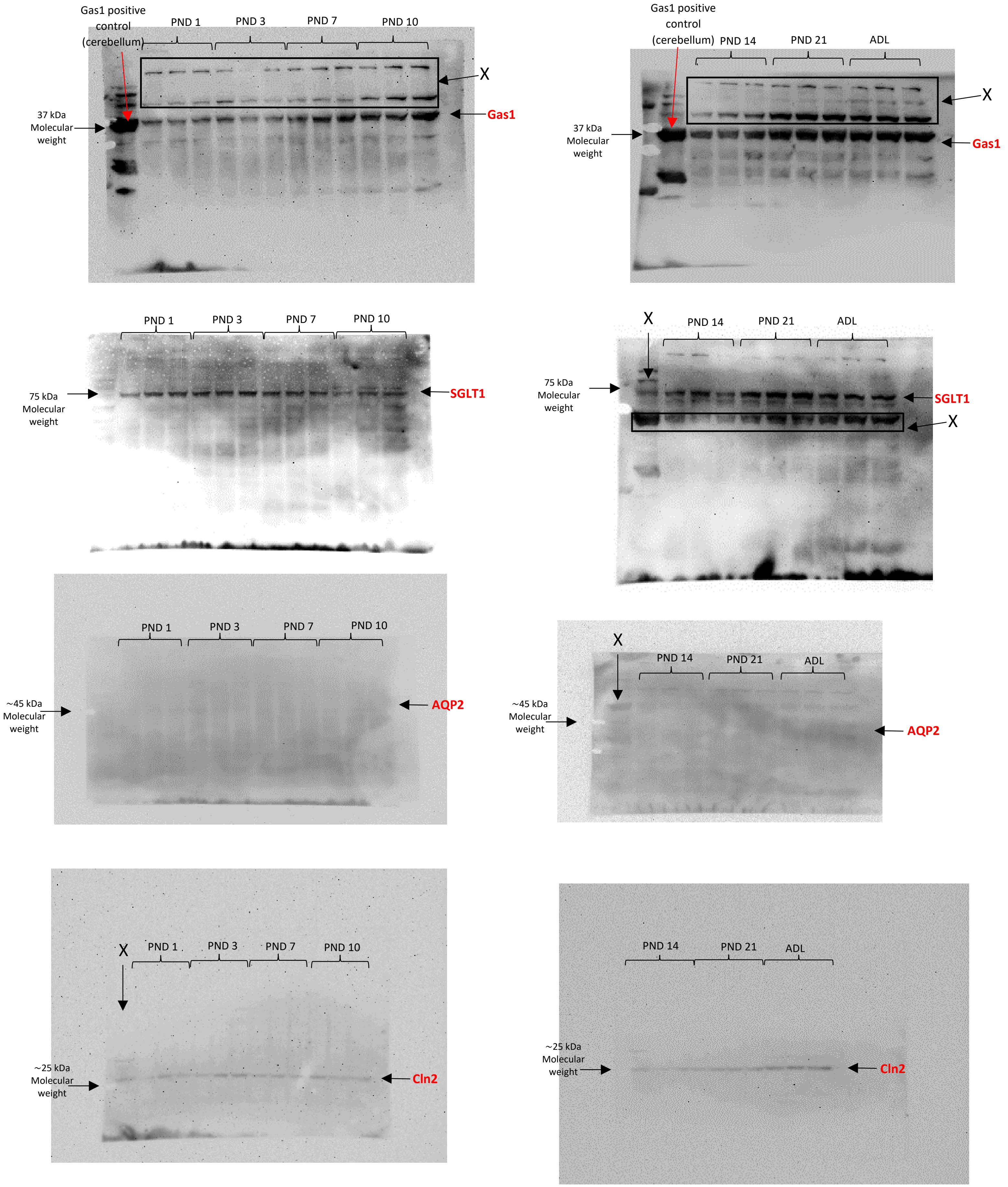

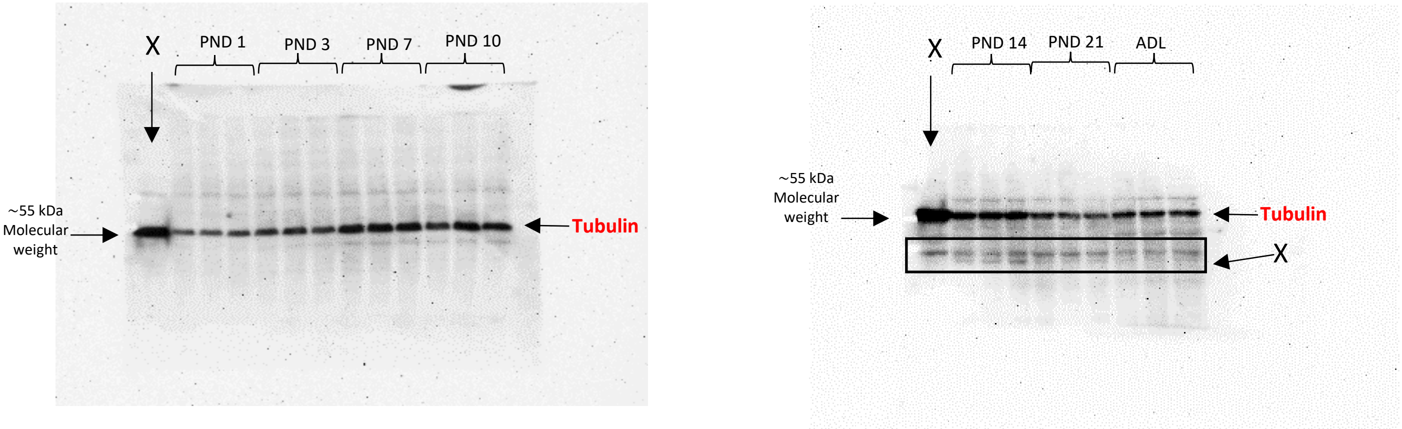

Figure 7 C. **Western blot of an enriched sample of distal tubules. Each PND analyzed is expressed as the relative density of a group of 20 (PND 1, 3, 7, 10 and 14) and 8 (21 PND and adult) rats normalized with actin as loading control.**

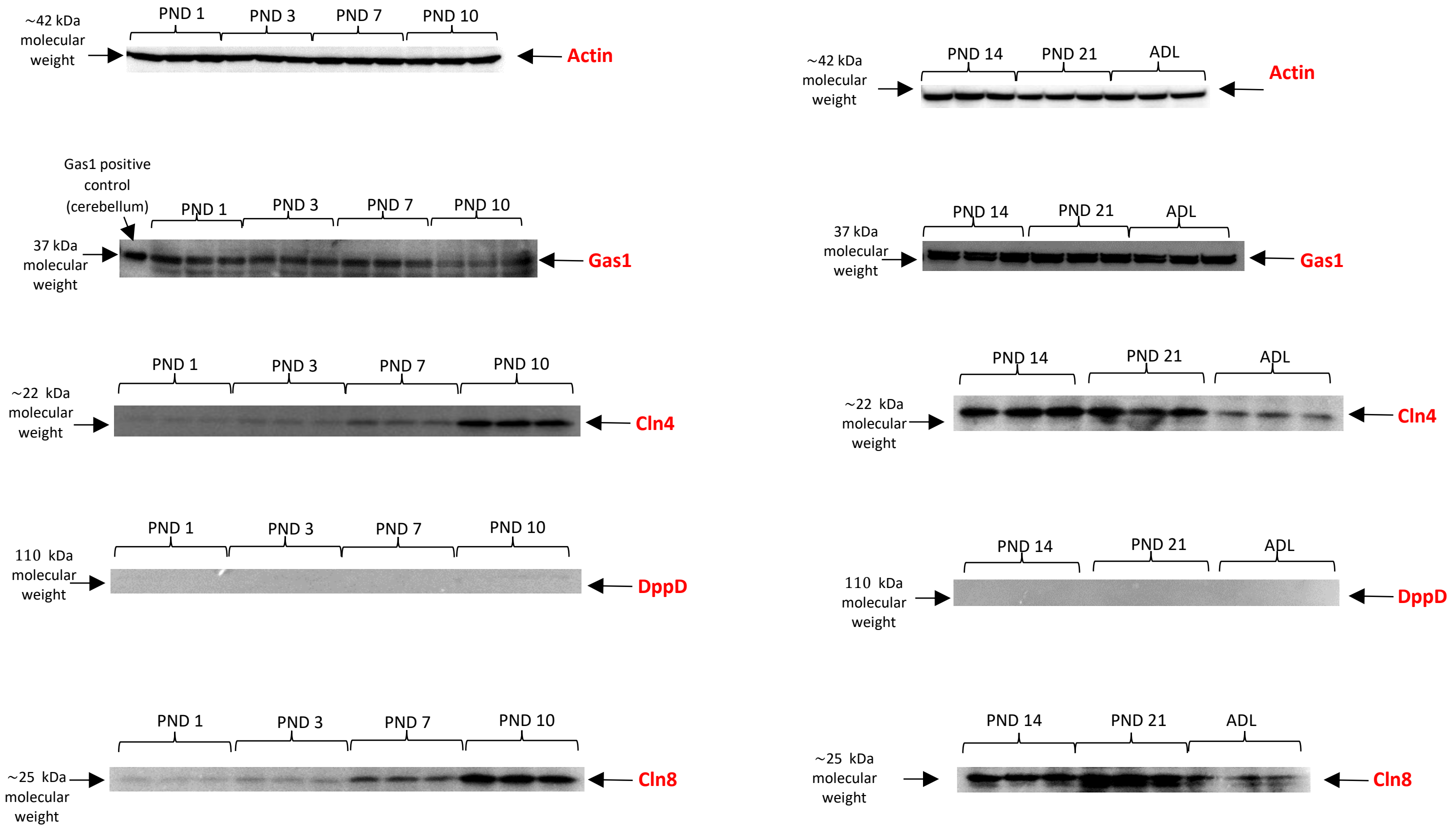

Supplement: S1 Raw images — (PDF) [file pone.0284816.s001.pdf]
